# Supplementary material for: Genetic loci determining potato starch yield and granule morphology revealed by genome-wide association study (GWAS)
Source: PeerJ. 2020 Nov 10;8:e10286. doi: 10.7717/peerj.10286 (PMC7664467; doi:10.7717/peerj.10286)
Supplement: Supplemental Information 2 [file peerj-08-10286-s002.docx]

**Table S1. Explanation and formulas for the morphological parameters of the starch granules micro images (https://imagej.nih.gov/ij/).**

| Name of the morphological parameter | Formula | Explanation |
| --- | --- | --- |
| Area | ---- | An area of the projection of the starch granule in the bright spot of the microscope. |
| Circularity | $4\pi\times\frac{\left[ Area \right]}{\left[ Perimeter \right]^{2}}$ | 1 – For ideal circle, 0 – for polygon. |
| Feret’s diameter | ---- | Correlates with Area |
| Min Feret’s diameter | ---- | ---- |
| Aspect ratio | $\frac{[Major Axis]}{[Minor Axis]}$ | 1 - For ideal curcle, ∞ - for linear shape |
| Roundness | $4\times\frac{[Area]}{\pi\times{[Major axis]}^{2}}$ | 4 - For ideal curcle, 0 - for linear shape. Correlates with Aspect ratio. |
| Solidity | $\frac{[Area]}{[Convex area]}$ | 1 – for ideal smooth surface, the closer to 0 the more uneven the surface is. |
