# Supplementary material for: Genetic loci determining potato starch yield and granule morphology revealed by genome-wide association study (GWAS)
Source: PeerJ. 2020 Nov 10;8:e10286. doi: 10.7717/peerj.10286 (PMC7664467; doi:10.7717/peerj.10286)
Supplement: Supplemental Information 3 [file peerj-08-10286-s003.docx]

**Table S2. “Phenotype – genotype” covariation of two blocks of principal components.**

| Axes | Bicomponents’ pair covariation | % |
| --- | --- | --- |
| **1** | **2.1683** | **45.764** |
| **2** | **1.9315** | **36.314** |
| **3** | **1.0425** | **10.578** |
| 4 | 0.7768 | 5.8738 |
| 5 | 0.32983 | 1.0589 |
| 6 | 0.17988 | 0.31498 |
| 7 | 0.090539 | 0.079795 |
| 8 | 0.041 | 0.016363 |
