# Supplementary material for: Genetic loci determining potato starch yield and granule morphology revealed by genome-wide association study (GWAS)
Source: PeerJ. 2020 Nov 10;8:e10286. doi: 10.7717/peerj.10286 (PMC7664467; doi:10.7717/peerj.10286)
Supplement: Supplemental Information 4 [file peerj-08-10286-s004.docx]

**Table S3. Correlation coefficients of studied traits with three bicomponents.**

| № | Traits | Correlation coefficients with bicomponents | | | | | |
| --- | --- | --- | --- | --- | --- | --- | --- |
|  |  | First bicomponent | | Second bicomponent | | Third bicomponent | |
|  |  | phenotypic | genotypic | phenotypic | genotypic | phenotypic | genotypic |
| 1 | Preparative yield of tuber starch | 0.63582 | 0.51109 | 0.24251 | 0.26905 | -0.6128 | -0.60307 |
| 2 | Aspect ratio | -0.44297 | -0.36608 | 0.83782 | 0.66041 | 0.10454 | 0.016328 |
| 3 | Area | 0.79301 | 0.56575 | 0.58495 | 0.42125 | 0.35082 | 0.14039 |
| 4 | Circularity | 0.67992 | 0.44457 | -0.58746 | -0.47097 | 0.0491 | 0.056704 |
| 5 | Feret’s diameter | 0.7675 | 0.53185 | 0.63433 | 0.4493 | 0.36934 | 0.14888 |
| 6 | Minimal Feret’s diameter | 0.85469 | 0.61039 | 0.48697 | 0.32829 | 0.34744 | 0.13842 |
| 7 | Roundness | 0.31949 | 0.30392 | -0.85731 | -0.67328 | -0.16438 | -0.05353 |
| 8 | Solidity | 0.83012 | 0.58126 | -0.29128 | -0.27433 | 0.15253 | 0.10696 |
