# Supplementary material for: Genetic loci determining potato starch yield and granule morphology revealed by genome-wide association study (GWAS)
Source: PeerJ. 2020 Nov 10;8:e10286. doi: 10.7717/peerj.10286 (PMC7664467; doi:10.7717/peerj.10286)
Supplement: Supplemental Information 5 [file peerj-08-10286-s005.docx]

**Table S4. Description of genes and their products (according to Plant.Essemble.com), associated with the protein-coding SNPs identified in the current study. (In case of several transcripts the one with higher molecular weight is shown).**

| **SNP-marker (nucleotide substitution)** | **Chr** | **Gene code** | **Gene statistics** | **Transcript name** | **Protein statistics** |
| --- | --- | --- | --- | --- | --- |
| **Preparative yield** | | | | | |
| PotVar0026637 (syn) | 4 | PGSC0003DMG400002859 | **Exons:** 2, **Coding exons:** 2, **Transcript length:** 762 bps, **Translation length:** 95 residues | Transcription regulator | Ave. residue weight: 111.872 g/mol  Charge: 0.5  Isoelectric point: 6.9818  Molecular weight: 10,627.88 g/mol  Number of residues: 95 aa |
| PotVar0098903 | 4 | - | - | - | - |
| PotVar0098904 | 4 | - | - | - | - |
| solcap_snp_c2_32042 (syn) | 4 | - | **-** | - | - |
| PotVar0034580 | 5 | PGSC0003DMG400023458 | **Exons:** 2, **Coding exons:** 2, **Transcript length:** 2,593 bps, **Translation length:** 707 residues | Phenylalanine ammonia-lyase | Ave. residue weight: 109.633 g/mol  Charge: -0.5  Isoelectric point: 6.4615  Molecular weight: 77,510.48 g/mol  Number of residues: 707 aa |
| solcap_snp_c2_52081 | 5 | PGSC0003DMG400000806 | **Exons:** 7, **Coding exons:** 7, **Transcript length:** 1,293 bps, **Translation length:** 301 residues | Coiled-coil domain-containing protein | Ave. residue weight: 112.574 g/mol  Charge: 22.5  Isoelectric point: 10.2503  Molecular weight: 33,884.80 g/mol  Number of residues: 301 aa |
| solcap_snp_c1_1250 (syn) | 5 | PGSC0003DMG400023319 | **Exons:** 8, **Coding exons:** 8, **Transcript length:** 1,653 bps, **Translation length:** 432 residues | Alcohol dehydrogenase | Ave. residue weight: 106.866 g/mol  Charge: 8.0  Isoelectric point: 7.7206  Molecular weight: 46,165.99 g/mol  Number of residues: 432 aa |
| solcap_snp_c2_3174 (syn) | 6 | PGSC0003DMG400002146 | **Exons:** 6, **Coding exons:** 6, **Transcript length:** 1,811 bps, **Translation length:** 495 residues | PTAC16 | Ave. residue weight: 106.186 g/mol  Charge: 6.0  Isoelectric point: 9.4991  Molecular weight: 52,562.09 g/mol  Number of residues: 495 aa |
| solcap_snp_c1_5970 | 7 | PGSC0003DMG400022216 | **Exons:** 1, **Coding exons:** 1, **Transcript length:** 1,687 bps, **Translation length:** 143 residues | SUI1 B protein | Ave. residue weight: 113.740 g/mol  Charge: 8.5  Isoelectric point: 9.9073  Molecular weight: 16,264.89 g/mol  Number of residues: 143 aa |
| PotVar0012073 (syn) | 9 | PGSC0003DMG400008953 | **Exons:** 5, **Coding exons:** 4, **Transcript length:** 1,168 bps, **Translation length:** 215 residues | Conserved gene of unknown function | Ave. residue weight: 110.201 g/mol  Charge: 10.5  Isoelectric point: 10.1393  Molecular weight: 23,693.29 g/mol  Number of residues: 215 aa |
| solcap_snp_c2_6285 | 11 | PGSC0003DMG400016242 | **Exons:** 12, **Coding exons:** 12, **Transcript length:** 1,901 bps, **Translation length:** 490 residues | Acetolactate synthase | Ave. residue weight: 108.665 g/mol  Charge: 10.5  Isoelectric point: 9.7432  Molecular weight: 53,246.07 g/mol  Number of residues: 490 aa |
| solcap_snp_c2_6309 (syn) | 11 | PGSC0003DMG400016238 | **Exons:** 8, **Coding exons:** 8, **Transcript length:** 2,108 bps, **Translation length:** 597 residues | DEGP10 | Ave. residue weight: 110.385 g/mol  Charge: 0.0  Isoelectric point: 6.5062  Molecular weight: 65,900.10 g/mol  Number of residues: 597 aa |
| PotVar0067347 (syn) | 11 | PGSC0003DMG400016168 | **Exons:** 11, **Coding exons:** 11, **Transcript length:** 2,146 bps, **Translation length:** 594 residues | Methylenetetrahydrofolate reductase | Ave. residue weight: 113.103 g/mol  Charge: -5.5  Isoelectric point: 5.5854  Molecular weight: 67,183.08 g/mol  Number of residues: 594 aa |
| solcap_snp_c2_6185 (syn) | 11 | PGSC0003DMG400007426 | **Exons:** 12, **Coding exons:** 12, **Transcript length:** 3,080 bps, **Translation length:** 880 residues | Conserved gene of unknown function | Ave. residue weight: 114.947 g/mol  Charge: -12.5  Isoelectric point: 5.4488  Molecular weight: 101,153.69 g/mol  Number of residues: 880 aa |
| solcap_snp_c2_5957 | 11 | PGSC0003DMG400016230 | **Exons:** 1, **Coding exons:** 1, **Transcript length:** 813 bps, **Translation length:** 202 residues | Conserved gene of unknown function | Ave. residue weight: 112.807 g/mol  Charge: 13.5  Isoelectric point: 10.8474  Molecular weight: 22,787.03 g/mol  Number of residues: 202 aa |
| **Aspect ratio and Roundness** | | | | | |
| PotVar0119913 | 1 | PGSC0003DMG400022782 | **Exons:** 14, **Coding exons:** 14, **Transcript length:** 1,749 bps, **Translation length:** 582 residues | Aspartokinase | Ave. residue weight: 110.035 g/mol  Charge: -14.0  Isoelectric point: 4.8831  Molecular weight: 64,040.45 g/mol  Number of residues: 582 aa |
| PotVar0071846 | 1 | PGSC0003DMT400081953 | **Exons:** 6, **Coding exons:** 6, **Transcript length:** 1,437 bps, **Translation length:** 297 residues | Oxoglutarate malate translocator | Ave. residue weight: 106.569 g/mol  Charge: 12.0  Isoelectric point: 9.7468  Molecular weight: 31,651.02 g/mol  Number of residues: 297 aa |
| solcap_snp_c2_36665 | 1 | PGSC0003DMG400019971 | **Exons:** 2, **Coding exons:** 2, **Transcript length:** 2,239 bps, **Translation length:** 634 residues | Circadian clock-associated FKF1 | Ave. residue weight: 111.020 g/mol  Charge: -21.0  Isoelectric point: 4.8031  Molecular weight: 70,386.59 g/mol  Number of residues: 634 aa |
| solcap_snp_c2_36659 | 1 | PGSC0003DMG400019971 | **Exons:** 2, **Coding exons:** 2, **Transcript length:** 2,239 bps, **Translation length:** 634 residues | Circadian clock-associated FKF1 | Ave. residue weight: 111.020 g/mol  Charge: -21.0  Isoelectric point: 4.8031  Molecular weight: 70,386.59 g/mol  Number of residues: 634 aa |
| PotVar0120075 | 1 | PGSC0003DMG400022778 | **Exons:** 4, **Coding exons:** 3, **Transcript length:** 3,271 bps, **Translation length:** 857 residues | Alpha,alpha-trehalose-phosphate synthase [UDP-forming] 6 | Ave. residue weight: 113.599 g/mol  Charge: -9.0  Isoelectric point: 5.6847  Molecular weight: 97,354.04 g/mol  Number of residues: 857 aa |
| PotVar0071852 | 1 | PGSC0003DMT400081953 | **Exons:** 6, **Coding exons:** 6, **Transcript length:** 1,437 bps, **Translation length:** 297 residues | Oxoglutarate malate translocator | Ave. residue weight: 106.569 g/mol  Charge: 12.0  Isoelectric point: 9.7468  Molecular weight: 31,651.02 g/mol  Number of residues: 297 aa |
| PotVar0119973 | 1 | PGSC0003DMG400022782 | **Exons:** 14, **Coding exons:** 14, **Transcript length:** 1,749 bps, **Translation length:** 582 residues | Aspartokinase | Ave. residue weight: 110.035 g/mol  Charge: -14.0  Isoelectric point: 4.8831  Molecular weight: 64,040.45 g/mol  Number of residues: 582 aa |
| solcap_snp_c2_36664 | 1 | PGSC0003DMG400019971 | **Exons:** 2, **Coding exons:** 2, **Transcript length:** 2,239 bps, **Translation length:** 634 residues | Circadian clock-associated FKF1 | Ave. residue weight: 111.020 g/mol  Charge: -21.0  Isoelectric point: 4.8031  Molecular weight: 70,386.59 g/mol  Number of residues: 634 aa |
| solcap_snp_c2_32254 | 2 | PGSC0003DMG400028032 | **Exons:** 6, **Coding exons:** 4, **Transcript length:** 3,159 bps, **Translation length:** 894 residues | WPP domain-associated protein | Ave. residue weight: 115.838 g/mol  Charge: -41.0  Isoelectric point: 4.7383  Molecular weight: 103,559.59 g/mol  Number of residues: 894 aa |
| solcap_snp_c2_14648 | 2 | PGSC0003DMG400002324 | **Exons:** 9, **Coding exons:** 8, **Transcript length:** 1,638 bps, **Translation length:** 393 residues | Plastid high chlorophyll fluorescence 136 | Ave. residue weight: 109.060 g/mol  Charge: 1.5  Isoelectric point: 6.9846  Molecular weight: 42,860.51 g/mol  Number of residues: 393 aa |
| solcap_snp_c2_48725 | 2 | PGSC0003DMG400017793 | **Exons:** 2, **Coding exons:** 1, **Transcript length:** 2,916 bps, **Translation length:** 705 residues | Pentatricopeptide repeat-containing protein | Ave. residue weight: 111.841 g/mol  Charge: -1.0  Isoelectric point: 6.4014  Molecular weight: 78,847.93 g/mol  Number of residues: 705 aa |
| solcap_snp_c1_3747 | 2 | PGSC0003DMG400014532 | **Exons:** 4, **Coding exons:** 4, **Transcript length:** 1,073 bps, **Translation length:** 259 residues | Conserved gene of unknown function | Ave. residue weight: 109.456 g/mol  Charge: 9.0  Isoelectric point: 9.8206  Molecular weight: 28,349.01 g/mol  Number of residues: 259 aa |
| solcap_snp_c1_16405 | 2 | PGSC0003DMG400014537 | **Exons:** 9, **Coding exons:** 9, **Transcript length:** 2,755 bps, **Translation length:** 745 residues | DNA binding protein | Ave. residue weight: 113.954 g/mol  Charge: 41.5  Isoelectric point: 9.6895  Molecular weight: 84,895.41 g/mol  Number of residues: 745 aa |
| solcap_snp_c1_3746 | 2 | PGSC0003DMG400014532 | **Exons:** 4, **Coding exons:** 4, **Transcript length:** 1,073 bps, **Translation length:** 259 residues | Conserved gene of unknown function | Ave. residue weight: 109.456 g/mol  Charge: 9.0  Isoelectric point: 9.8206  Molecular weight: 28,349.01 g/mol  Number of residues: 259 aa |
| solcap_snp_c2_4353 | 2 | - | - | - | - |
| PotVar0032402 | 2 | - | - | - | - |
| solcap_snp_c2_57190 | 2 | - | - | - | - |
| PotVar0032114 | 2 | - | - | - | - |
| solcap_snp_c2_4354 | 2 | - | - | - | - |
| solcap_snp_c2_4360 | 2 | - | - | - | - |
| solcap_snp_c2_14652 | 2 | - | - | - | - |
| solcap_snp_c1_16379 | 2 | - | - | - | - |
| solcap_snp_c1_1503 | 2 | - | - | - | - |
| solcap_snp_c2_48735 | 2 | - | - | - | - |
| solcap_snp_c1_3750 | 2 | - | - | - | - |
| solcap_snp_c2_56617 | 2 | - | - | - | - |
| PotVar0032432 | 2 | - | - | - | - |
| PotVar0022442 | 7 | PGSC0003DMG400011200 | **Exons:** 7, **Coding exons:** 6, **Transcript length:** 2,642 bps, **Translation length:** 596 residues | Conserved gene of unknown function | Ave. residue weight: 110.259 g/mol  Charge: 44.0  Isoelectric point: 11.0298  Molecular weight: 65,714.56 g/mol  Number of residues: 596 aa |
| solcap_snp_c2_33657 | 11 | PGSC0003DMG400016200 | **Exons:** 2, **Coding exons:** 2, **Transcript length:** 1,769 bps, **Translation length:** 172 residues | Conserved gene of unknown function | Ave. residue weight: 114.546 g/mol  Charge: -5.0  Isoelectric point: 4.7889  Molecular weight: 19,701.86 g/mol  Number of residues: 172 aa |
| solcap_snp_c2_50824 | 12 | - | - | - | - |
| solcap_snp_c1_1504 | 2 | - | - | - | - |
| solcap_snp_c2_54815 | 1 | - | **-** | - | - |
| **Circularity** | | | | | |
| solcap_snp_c1_15462 | 7 | PGSC0003DMG400011837 | **Exons:** 18, **Coding exons:** 18, **Transcript length:** 2,634 bps, **Translation length:** 737 residues | Minichromosome maintenance 5 protein | Ave. residue weight: 111.199 g/mol  Charge: 8.0  Isoelectric point: 7.6321  Molecular weight: 81,953.37 g/mol  Number of residues: 737 aa |
| PotVar0047235 | 11 | PGSC0003DMT400001124 | **Exons:** 3, **Coding exons:** 3, **Transcript length:** 1,709 bps, **Translation length:** 447 residues | Flavonoid 3',5'-hydroxylase | Ave. residue weight: 112.695 g/mol  Charge: 4.0  Isoelectric point: 7.3993  Molecular weight: 50,374.51 g/mol  Number of residues: 447 aa |
| solcap_snp_c2_50824 | 12 | - | **-** | - | - |
| **1^st^ bi-component** | | | | | |
| solcap_snp_c2_52067 | 5 | PGSC0003DMG400000803 | **Exons:** 4, **Coding exons:** 4, **Transcript length:** 1,133 bps, **Translation length:** 242 residues | Cytochrome B561 family protein | Ave. residue weight: 111.264 g/mol  Charge: 15.0  Isoelectric point: 9.9658  Molecular weight: 26,925.91 g/mol  Number of residues: 242 aa |
| solcap_snp_c2_52081 | 5 | PGSC0003DMG400000806 | **Exons:** 7, **Coding exons:** 7, **Transcript length:** 1,293 bps, **Translation length:** 301 residues | Coiled-coil domain-containing protein | Ave. residue weight: 112.574 g/mol  Charge: 22.5  Isoelectric point: 10.2503  Molecular weight: 33,884.80 g/mol  Number of residues: 301 aa |
| solcap_snp_c1_15462 | 7 | PGSC0003DMG400011837 | **Exons:** 18, **Coding exons:** 18, **Transcript length:** 2,634 bps, **Translation length:** 737 residues | Minichromosome maintenance 5 protein | Ave. residue weight: 111.199 g/mol  Charge: 8.0  Isoelectric point: 7.6321  Molecular weight: 81,953.37 g/mol  Number of residues: 737 aa |
| PotVar0097065 | 9 | PGSC0003DMT400042818 | **Exons:** 19, **Coding exons:** 19, **Transcript length:** 4,195 bps, **Translation length:** 1,202 residues | Glucan/water dikinase | Ave. residue weight: 109.995 g/mol  Charge: -8.0  Isoelectric point: 5.9483  Molecular weight: 132,214.18 g/mol  Number of residues: 1,202 aa |
| PotVar0097020 | 9 | PGSC0003DMT400042818 | **Exons:** 19, **Coding exons:** 19, **Transcript length:** 4,195 bps, **Translation length:** 1,202 residues | Glucan/water dikinase | Ave. residue weight: 109.995 g/mol  Charge: -8.0  Isoelectric point: 5.9483  Molecular weight: 132,214.18 g/mol  Number of residues: 1,202 aa |
| PotVar0052560 | 12 | PGSC0003DMG400004687 | **Exons:** 8, **Coding exons:** 8, **Transcript length:** 1,197 bps, **Translation length:** 398 residues | Pto-interacting protein 1 | Ave. residue weight: 108.637 g/mol  Charge: 9.5  Isoelectric point: 8.2491  Molecular weight: 43,237.33 g/mol  Number of residues: 398 aa |

Syn – synonymous.
