# Supplementary figures and images for: Genetic loci determining potato starch yield and granule morphology revealed by genome-wide association study (GWAS)
Source: PeerJ. 2020 Nov 10;8:e10286. doi: 10.7717/peerj.10286 (PMC7664467; doi:10.7717/peerj.10286)

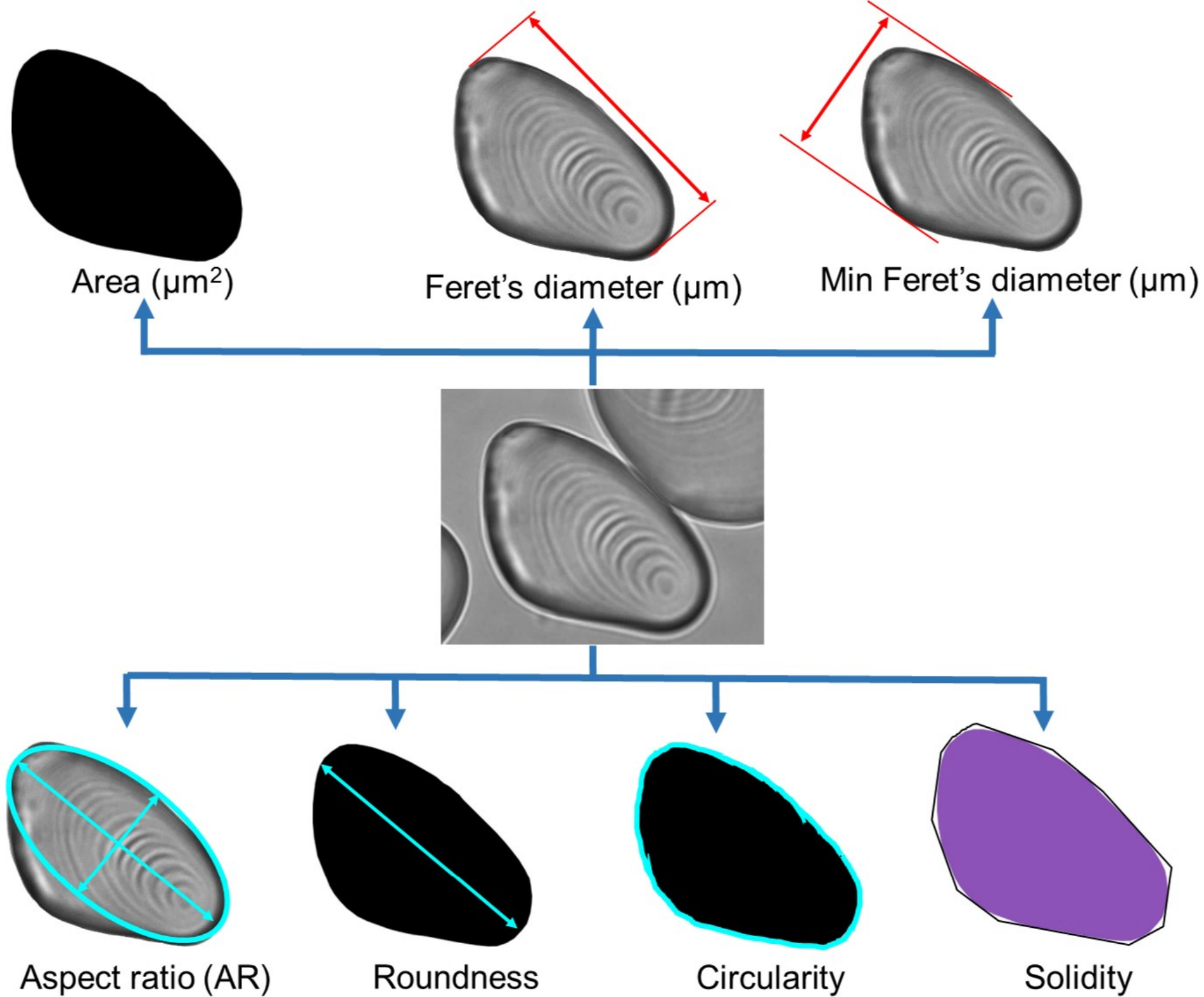

Supplement: Supplemental Information 6 [file peerj-08-10286-s006.pdf]

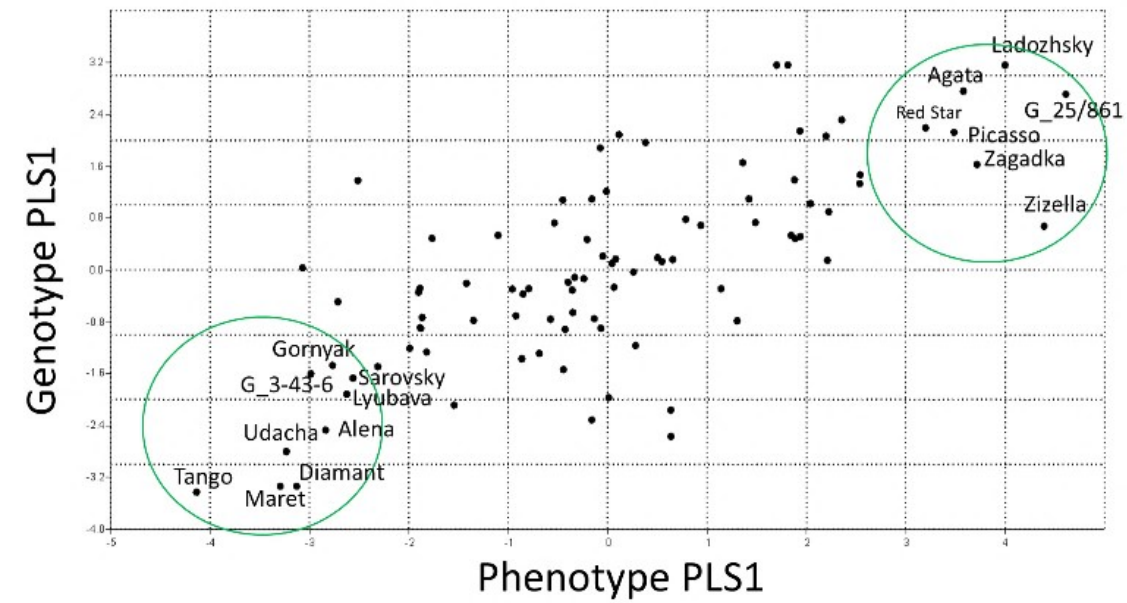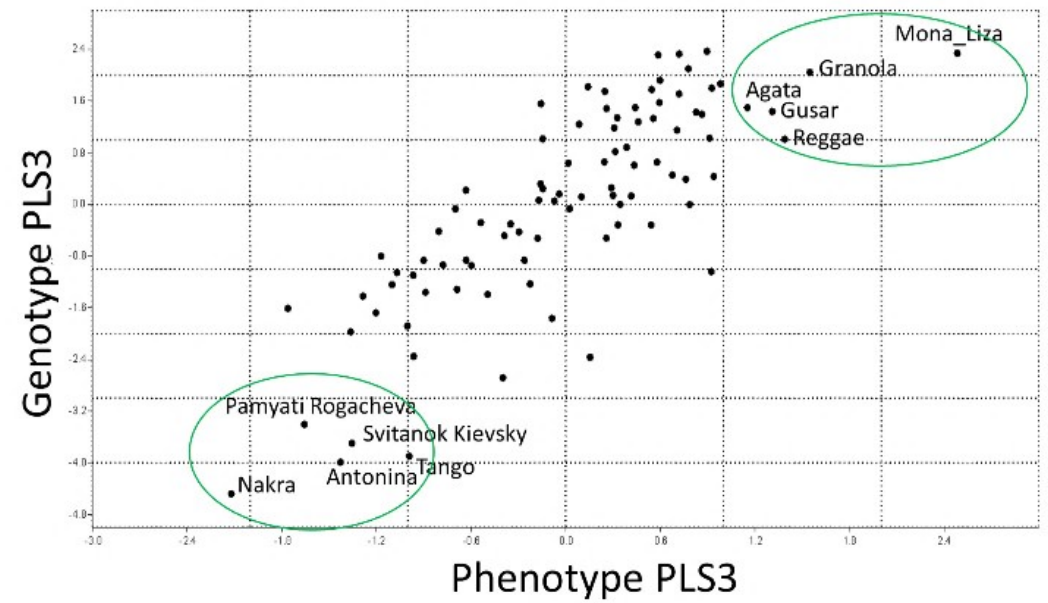

Supplement: Supplemental Information 7 [file peerj-08-10286-s007.pdf]

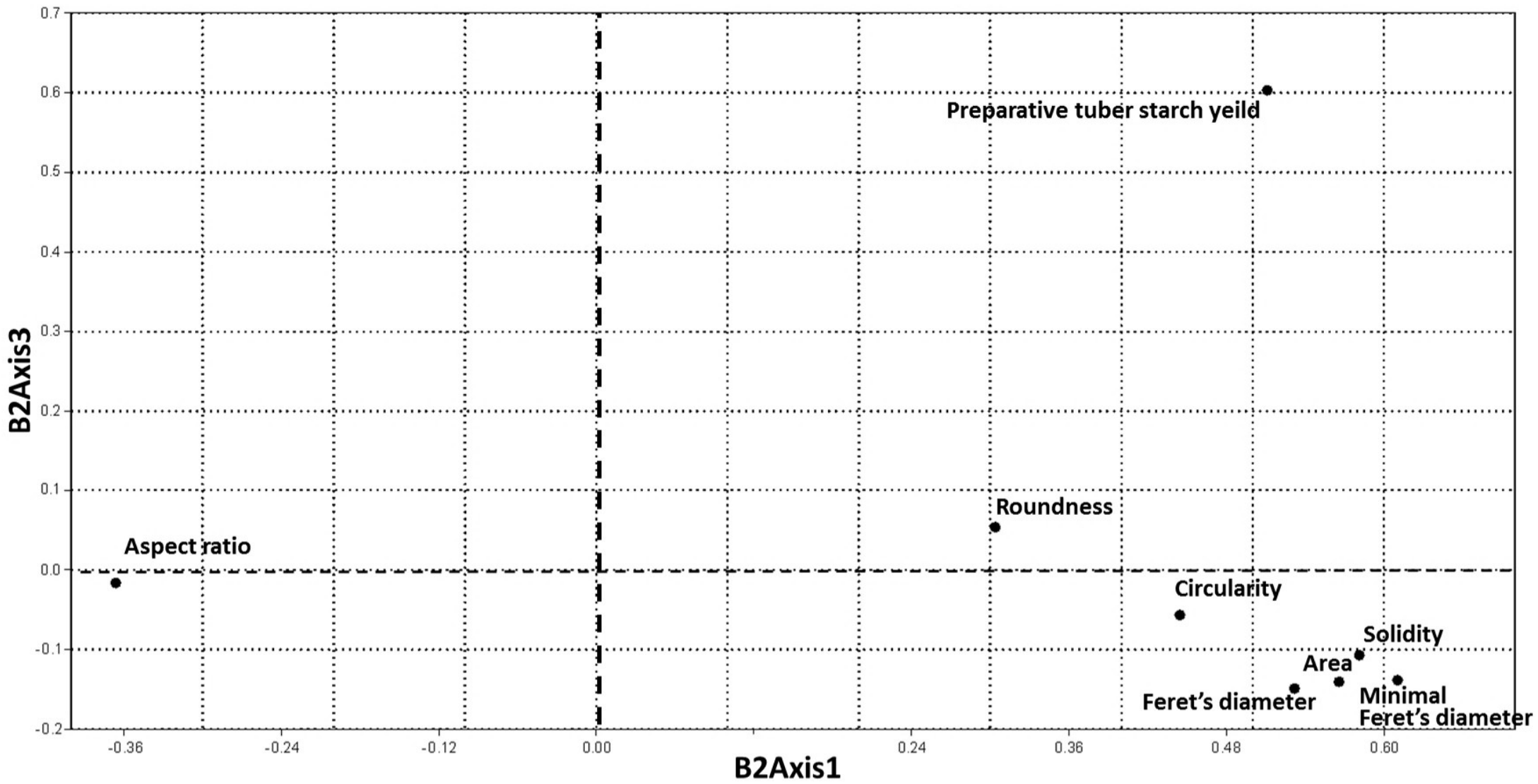

Supplement: Supplemental Information 9 [file peerj-08-10286-s009.pdf]

$p$ -value: 0.022

$2.3 \times 10^{-10}$

$1.8 \times 10^{-62}$

$1.07 \times 10^{-29}$

$1.83 \times 10^{-11}$

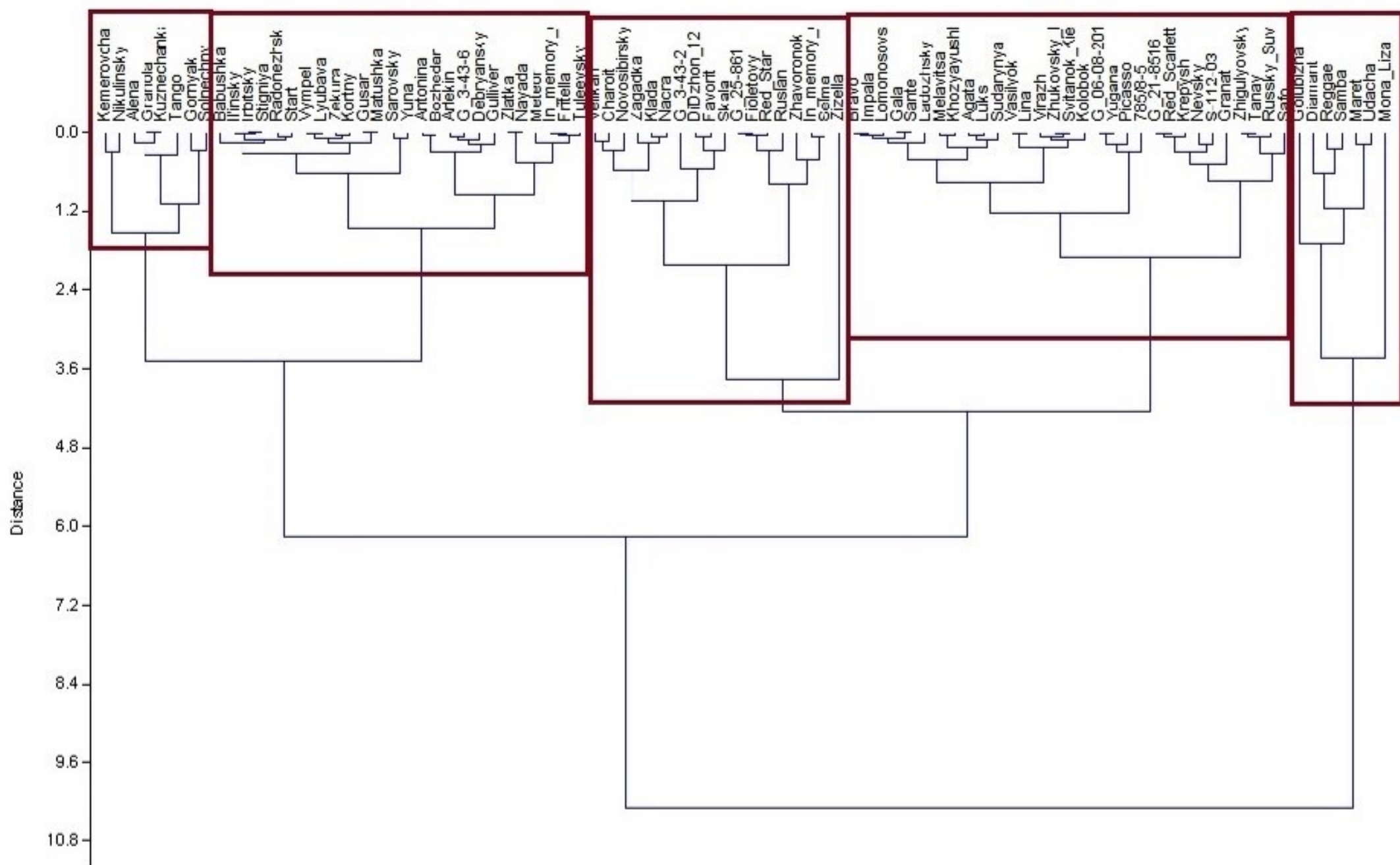

Supplement: Supplemental Information 14 [file peerj-08-10286-s014.pdf]

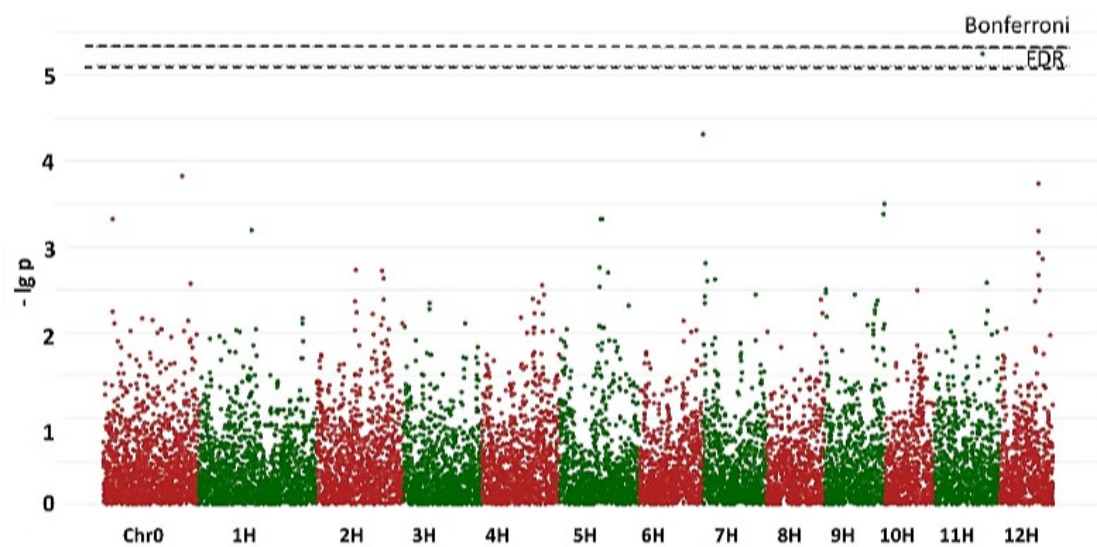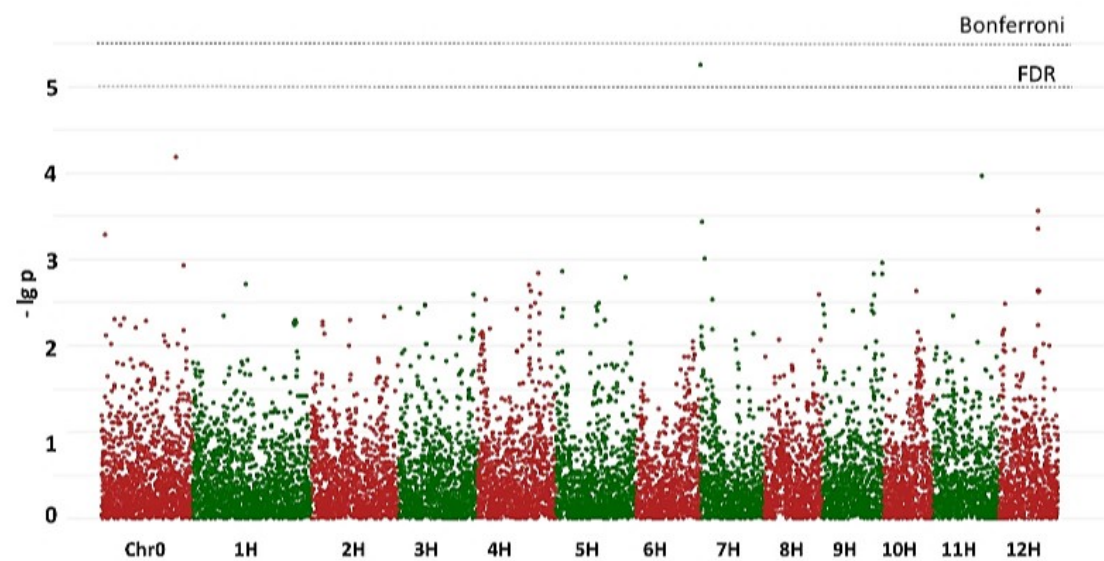

Supplement: Supplemental Information 15 [file peerj-08-10286-s015.pdf]

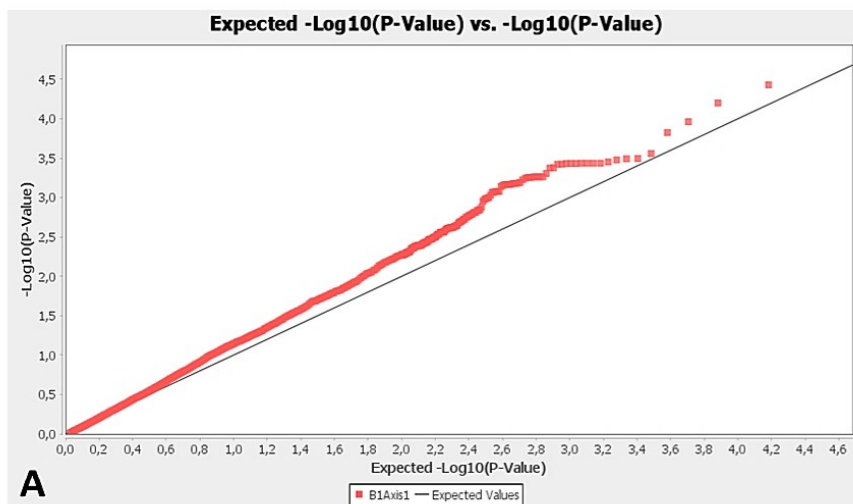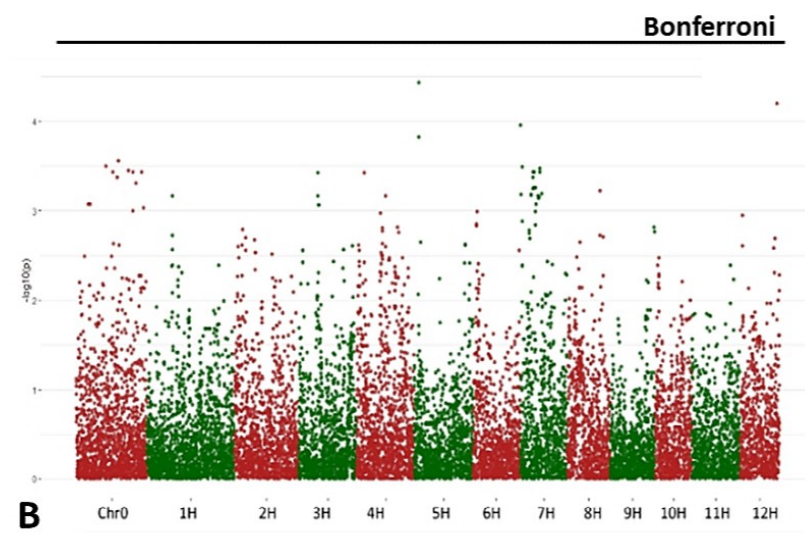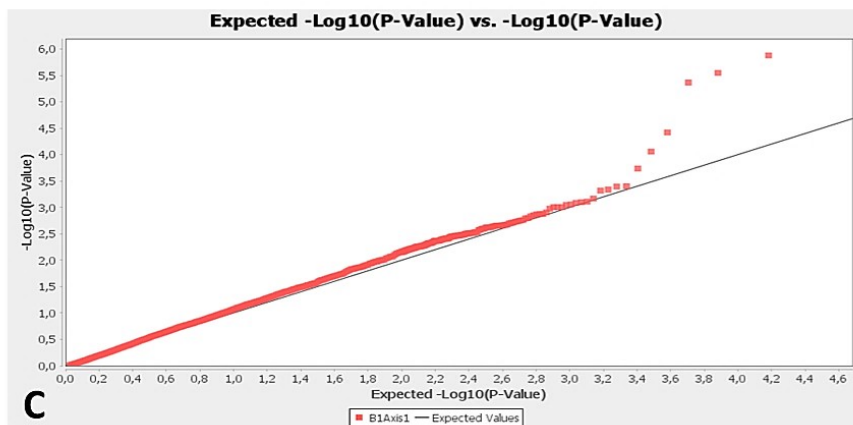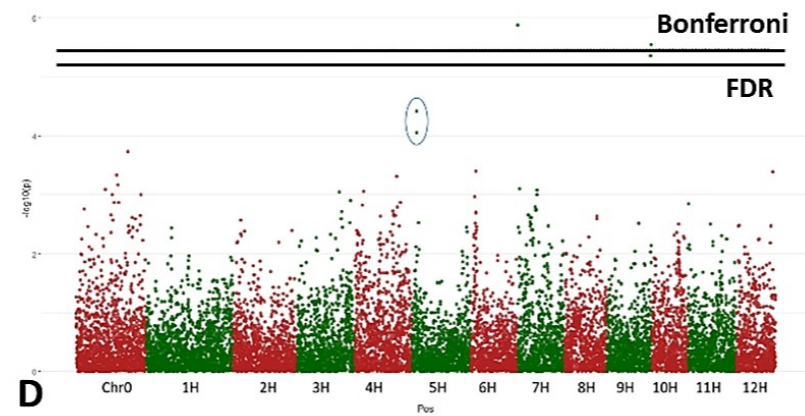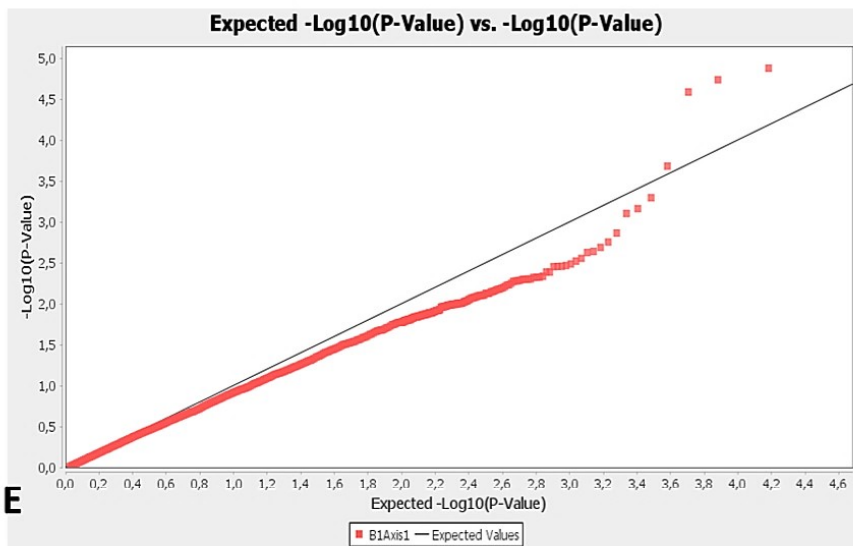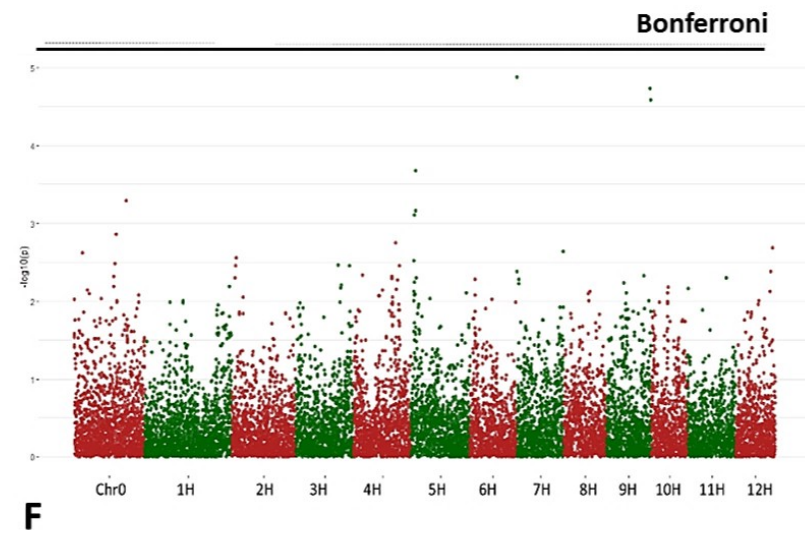

Supplement: Supplemental Information 16 [file peerj-08-10286-s016.pdf]
