# Supplementary material for: Genetic loci determining potato starch yield and granule morphology revealed by genome-wide association study (GWAS)
Source: PeerJ. 2020 Nov 10;8:e10286. doi: 10.7717/peerj.10286 (PMC7664467; doi:10.7717/peerj.10286)
Supplement: Supplemental Information 12 [file peerj-08-10286-s012.pdf]

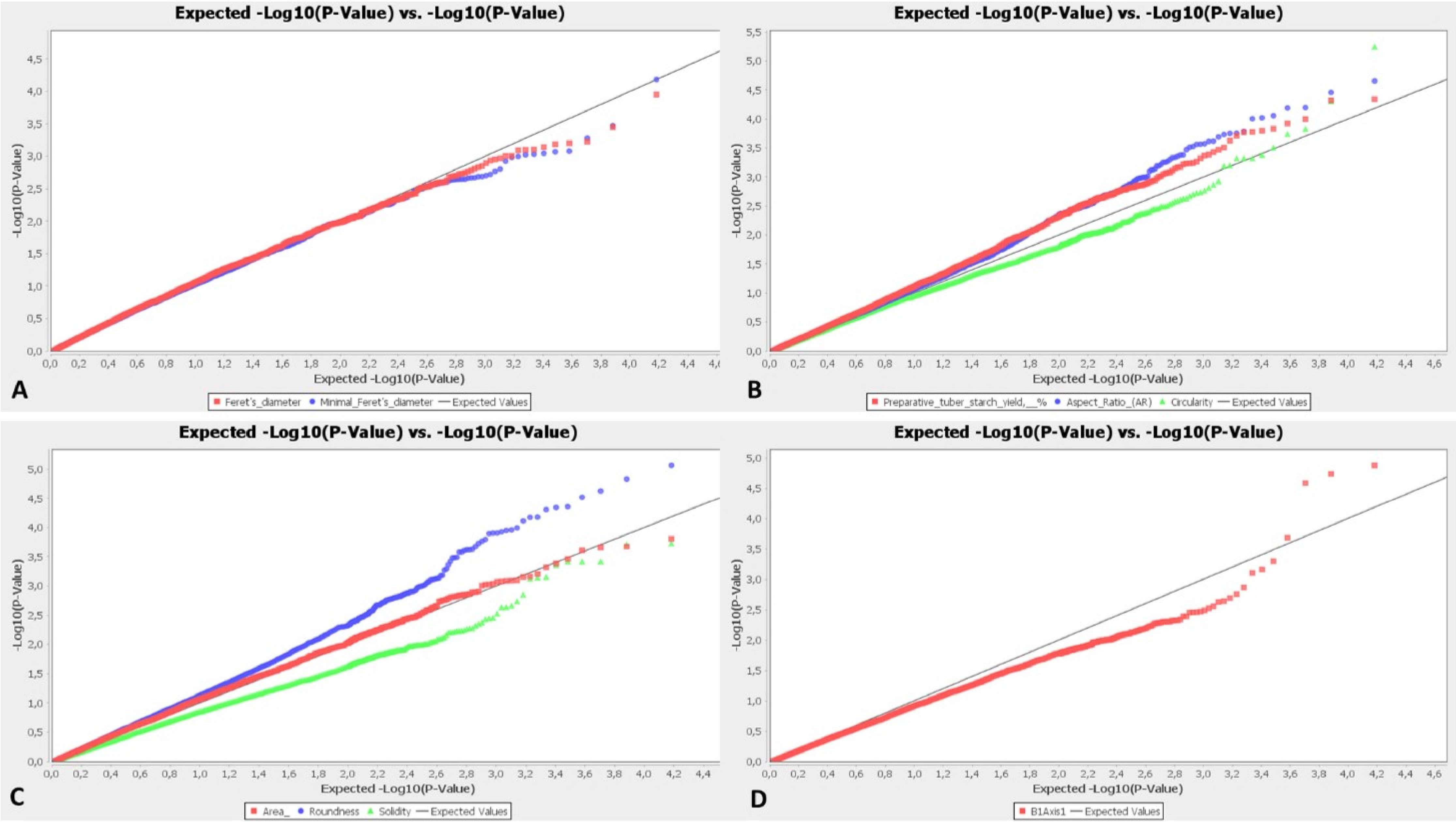
Expected -Log10(P-Value) vs. -Log10(P-Value)
-Log10(P-Value)
Expected -Log10(P-Value)
A
Feret's_diameter
Minimal_Feret's_diameter
Expected Values
Expected -Log10(P-Value) vs. -Log10(P-Value)
-Log10(P-Value)
Expected -Log10(P-Value)
B
Preparative_tuber_starch_yield,__%
Aspect_Ratio_(AR)
Circularity
Expected Values
Expected -Log10(P-Value) vs. -Log10(P-Value)
-Log10(P-Value)
Expected -Log10(P-Value)
C
Area_
Roundness
Solidity
Expected Values
Expected -Log10(P-Value) vs. -Log10(P-Value)
-Log10(P-Value)
Expected -Log10(P-Value)
D
B1Axis1
Expected Values
