# Supplementary material for: Genetic loci determining potato starch yield and granule morphology revealed by genome-wide association study (GWAS)
Source: PeerJ. 2020 Nov 10;8:e10286. doi: 10.7717/peerj.10286 (PMC7664467; doi:10.7717/peerj.10286)
Supplement: Supplemental Information 8 [file peerj-08-10286-s008.pdf]

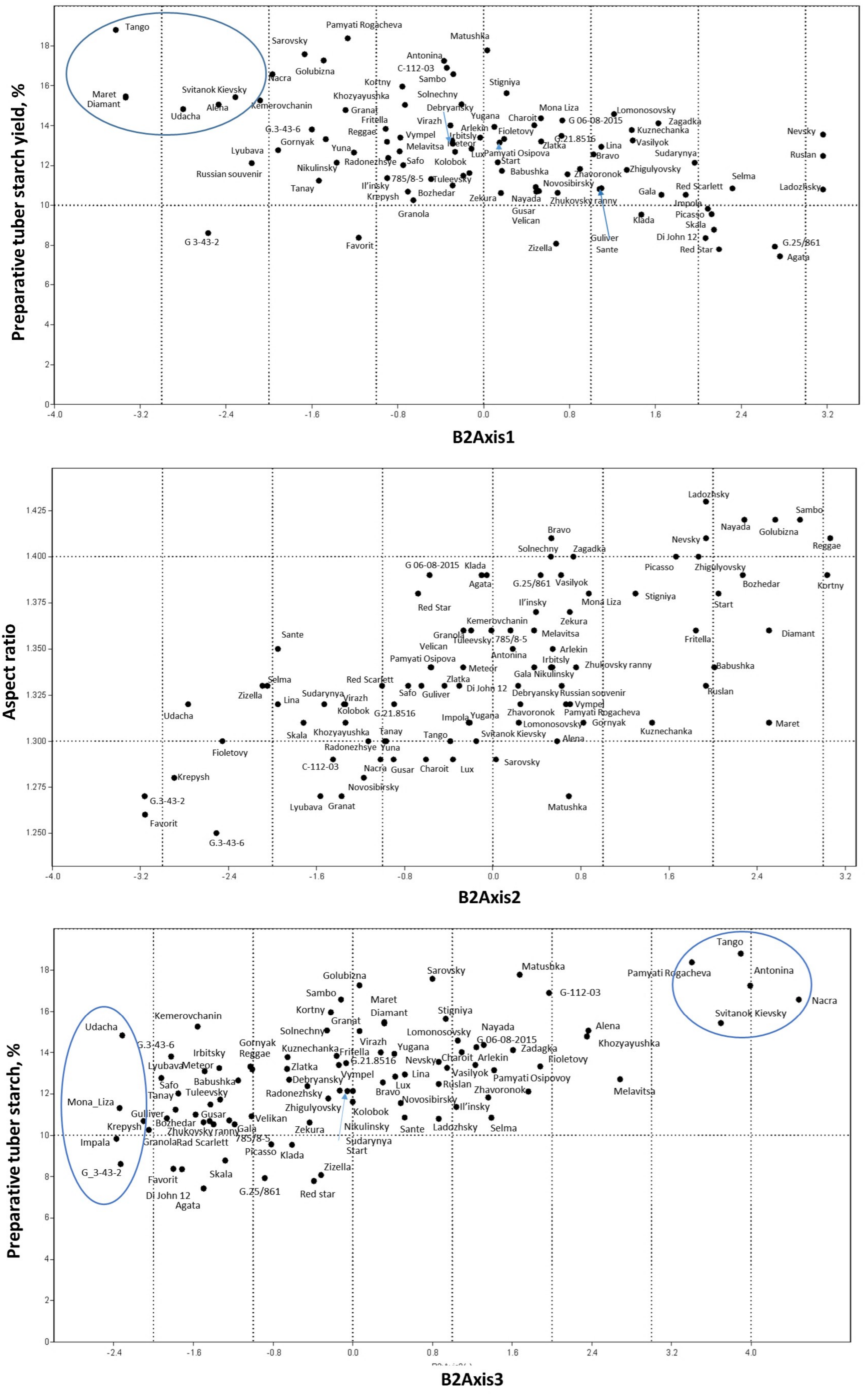

Preparative tuber starch yield, %
B2Axis1
Tango
Maret
Diamant
Udacha
Alena
Svitanok Kievsky
Kemerovchanin
Nacra
Sarovsky
Golubizna
Pamyati Rogacheva
Matushka
Antonina
C-112-03
Sambo
Kortny
Khozyayushka
Granat
Solnechny
Debryansky
Stigniya
Lyubava
Russian souvenir
G.3-43-6
Gornyak
Yuna
Nikulinsky
Tanay
Radonezhsye
Fritella
Reggae
Vympel
Melavitsa
Safo
785/8-5
Il'insky
Krepysh
Bozhedar
Granola
Tuleevsky
Kolobok
Virazh
Irbitsly
Meteor
Arlekin
Lux
Yugana
Pamyati Osipova
Start
Fioletovy
Charoit
Mona Liza
G 06-08-2015
G.21.8516
Zlatka
Babushka
Zekura
Nayada
Gusar
Velican
Novosibirsky
Zhukovsky ranny
Zhavoronok
Lina
Bravo
Lomonosovsky
Zagadka
Kuznechanka
Vasilyok
Zhigulyovsky
Sudarynya
Gala
Klada
Red Scarlett
Impola
Picasso
Skala
Di John 12
Red Star
Selma
Zizella
Guliver
Sante
G 3-43-2
Favorit
Nevsky
Ruslan
Ladozhsky
G.25/861
Agata
Aspect ratio
B2Axis2
Preparative tuber starch, %
B2Axis3
Mona_Liza
Impala
G_3-43-2
G-112-03
Rad Scarlett
Pamyati Osipovoy
